# MiR-6803-5p Promotes Cancer Cell Proliferation and Invasion via PTPRO/NF-*κ*B Axis in Colorectal Cancer

**DOI:** 10.1155/2019/8128501

**Published:** 2019-11-20

**Authors:** Shushan Yan, Min Cheng, Quanhong Duan, Zengfang Wang, Wenfeng Gao, Bin Ren, Donghua Xu

**Affiliations:** ^1^Department of Gastrointestinal and Anal Diseases Surgery, the Affiliated Hospital of Weifang Medical University, Weifang 261000, China; ^2^Department of Physiology, Weifang Medical University, Weifang 261000, China; ^3^Department of Gynecology and Obstetrics, Weifang Hospital of Maternal and Child Health, Weifang 261000, China; ^4^Department of Rheumatology, Affiliated Hospital of Weifang Medical University & Clinical Medicine College, Weifang Medical University, Weifang 261000, China

## Abstract

Accumulated studies have implicated microRNAs (miRNAs) exert modifying effects on colorectal cancer (CRC). Protein tyrosine phosphatase, receptor type O (PTPRO) has been identified as a tumor suppressor in several kinds of cancer, including CRC. Previously, we have found that exosome-encapsulated miR-6803-5p is increased in CRC. However, the mechanism of miR-6803-5p in CRC is not clear yet. This study is aimed at elucidating the effect of miR-6803-5p in colorectal carcinogenesis. Expression of miR-6803-5p and PTPRO mRNA in peripheral blood mononuclear cells of CRC patients is estimated by real-time PCR. PTPRO protein in CRC cells is detected by western blot. To verify the association of miR-6803-5p with PTPRO, luciferase reporter assay is performed. CCK-8 and EdU assays are conducted to assess cell proliferation. Real-time PCR and ELISA are applied to detect cytokine expression in CRC cells. Cell invasion and migration assays are evaluated by transwell and scratch tests. Immunofluorescence is carried out to determine the activation of NF-*κ*B in HCT116 cells. Negative correlation is demonstrated between miR-6803-5p and PTPRO in CRC. PTPRO is demonstrated to be a direct target of miR-6803-5p. miR-6803-5p can promote cancer cell proliferation and invasion and enhance inflammation through PTPRO/NF-*κ*B axis in CRC, which serves as a useful target for CRC.

## 1. Introduction

Colorectal cancer (CRC) is a common malignant disease across the world. It has been well established that environmental factors, genetics, and interactions between them are closely associated with colorectal carcinogenesis [1–3]. However, the etiology and pathogenesis of CRC are not very clear yet. Protein tyrosine phosphatase, receptor type O (PTPRO) belongs to the R3 subtype family of receptor-type protein tyrosine phosphatase. It has been suggested as a tumor suppressor for multiple malignancies, such as CRC, hepatocellular carcinoma, and breast cancer [4–6]. PTPRO plays a critical role in regulating cancer inflammation and tumor immunity. Nevertheless, little is known about the precise molecular mechanism of PTPRO in CRC.

Many studies have suggested the important role of noncoding RNAs in colorectal carcinogenesis, which are implicated as promising biomarkers in many kinds of malignancies [7, 8]. In spite of not coding proteins, dysregulated noncoding RNAs have offered promising strategies for CRC diagnosis and prognosis [9]. microRNAs (miRNAs) have 20-24 nucleotides in length. They participate in regulating a variety of genes at the posttranscriptional level by influencing the translation of messenger RNAs (mRNAs) [10–12]. miRNAs have been established to influence multiple biological activities, including cancer cell proliferation, invasion, migration, and epithelial mesenchymal transition (EMT) [13–15]. They act either as oncogenes or tumor suppressors in cancer. Increasing evidence has suggested that extracellular vesicle miRNAs are capable of mediating cell-to-cell communications by transferring bioactive molecules [15–17]. Extracellular vesicles consist of microvesicles, exosomes, and apoptotic bodies, which are essential for intercellular communications within the complicated tumor microenvironment [17, 18]. During the past few years, investigations of miRNAs have made miRNAs useful markers for the diagnosis and therapy of cancer. miR-6803-5p is a newly found noncoding RNA in cancer [19, 20]. We have previously found that the exosome-encapsulated miR-6803-5p is obviously elevated in CRC [19]. Exosome-derived miR-6803-5p can also affect the progression and survival of CRC [19]. However, the mechanism of miR-6803-5p in CRC pathogenesis remains unknown. The object of the present study is to elucidate the effect of miR-6803-5p and its mechanism in CRC by a series of studies.

## 2. Materials and Methods

### 2.1. Patients and Samples

Our study is supervised by the Institutional Ethics Committee. Peripheral blood mononuclear cells (PBMCs) are isolated from 95 cases and 80 healthy controls adjusted by age and sex, who are registered in the same hospital at the same time. Before experiments, the informed consent has been signed by patients and controls. Characteristics of cases and controls are presented in Table 1.

### 2.2. Cell Line

HCT116 cell line is maintained in fresh Dulbecco's Modified Eagle's Medium (DMEM) (Invitrogen, USA) with 10% FBS (Gibco, USA). The DMEM is supplemented with penicillin/streptomycin and L-glutamine. Lentivirus plasmids are applied to transfect HCT116 cells (0.5 × 10^6^/well) and make cells overexpressed of miR-6803-5p and/or PTPRO.

### 2.3. Enzyme-Linked Immunosorbent Assay (ELISA)

TNF-*α* and IL-6 in the cultural supernatant are detected by ELISA using human ELISA kits (Dakewe Biotech, China). Details are presented in the published study [21].

### 2.4. Real-Time Polymerase Chain Reaction (PCR)

Trizol (Invitrogen, Paisley, UK) is adopted to purify total RNAs from PBMCs or HCT116 cells treated for 12 hours. We use 0.5 *μ*g total RNAs for the synthesis of cDNAs. Besides, we apply the Takara kit of SYBR Premix Ex Taq (Dalian, China) for PCR.  Table 2 shows the sequences of all primers.

### 2.5. Western Blot

1 × 10^6^ HCT116 cells are incubated in a 6-well plate under conditions of serum-free DMEM overnight. Then, plasmids of lv-miR-6803-5p, lv-PTPRO, and the corresponding control (lv-control) are used to transfect cells for 48 hours. Western blot analysis is performed after transfection. Primary antibodies of PTPRO (Proteintech Group), p-NF-*κ*B (CST, USA), E-cadherin (CST, USA), Vimentin (CST, USA), *β*-actin (CST, USA), and GAPDH (Sigma, MO, USA) are used for determination after cells are treated for 12 hours.

### 2.6. Luciferase Reporter Assay

In this study, 3 × 10^4^ HCT116 cells are cultured in 24-well plates in serum-free DMEM for 12 h. Cells are transfected with the lv-control and lv-miR-6803-5p plasmid, with the Renilla luciferase gene (Promega, Madison, USA) as internal normalization, and constructs containing PTPRO 3′UTR and the corresponding mutants for 36 h. Luciferase activity is assayed for analysis.

### 2.7. Cell Proliferation Assay

The proliferation of HCT116 cells (lv-control and lv-miR-6803-5p) is estimated by CCK-8 kit (Dojindo, Japan) when cells are incubated for 12, 24, and 48 hours based on the instructions. EdU assay is also conducted as previously described to estimate cancer cell proliferation [21].

### 2.8. Cell Invasion and Migration Assay

Transwell and scratch assays are carried out to estimate the invasion and migration of HCT116 cancer cells, respectively. In brief, we percolate transwell plates (Corning Costar, MA, USA) by use of Matrigel (BD Biosciences, NY, USA) for subsequent tests. About 2 × 10^4^ cells per well are incubated at the upper compartment in serum-free DMEM. We add DMEM with 10% FBS into the lower chamber as chemotaxin. After 12 hours, the filters are stained with 0.1% crystal violet after fixed with 4% paraformaldehyde. Scratch analysis is performed based on the protocol of reagents when HCT116 cancer cells are incubated in serum-free DMEM for 12 hours. The status of HCT116 cell invasion and migration is observed under the microscope. Experiments are, respectively, performed three times.

### 2.9. Immunofluorescence

The phosphorylation and activation of NF-*κ*B in CRC cells is detected by immunofluorescence as described in a previously published study [21]. In brief, the antibody of p-NF-*κ*B/P65 (CST, USA) and the secondary antibody labeled with PE (CST, USA) are used for determination. The intensity of cell fluorescence is scanned by confocal laser scanning microscope.

### 2.10. Statistical Analysis

We apply Student's *t*-test or one-way ANOVA to estimate the data by using SPSS 13.0 and GraphPad Prism 4.0. Two-tailed *P* < 0.05 is significant for all analyses. We carry out experiments for three times for each determination.

## 3. Results

### 3.1. miR-6803-5p and PTPRO Are Dysregulated in CRC

**Figure 1:**
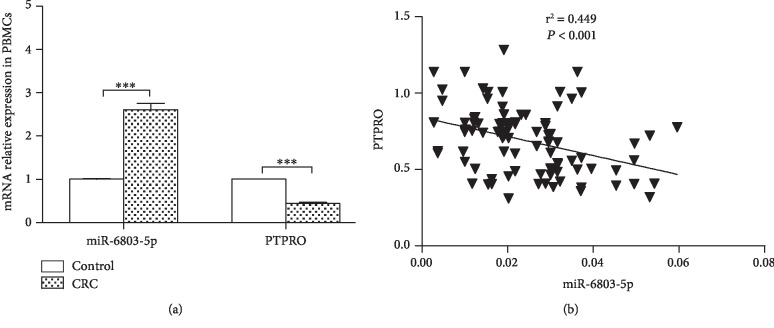
miR-6803-5p and PTPRO expression and their association in CRC. miR-6803-5p and PTPRO expression in PBMCs of cases and controls (cases/controls: 95/80; ^∗∗∗^*P* < 0.001). miR-6803-5p was negatively correlated with PTPRO.

Increased miR-6803-5p expression is found in PBMCs of CRC patients (Figure1(a)). However, PTPRO mRNA in PBMCs of cases is significantly reduced when comparing with controls (Figure1(a)). Interestingly, negative relationship is demonstrated between miR-6803-5p and PTPRO regarding the expression in PBMCs from CRC cases (Figure1(b)). Accordingly, we hypothesize that miR-6803-5p may affect the pathogenesis of CRC by regulating PTPRO at the posttranscriptional level.

### 3.2. miR-6803-5p Targetedly Regulates PTPRO

**Figure 2:**
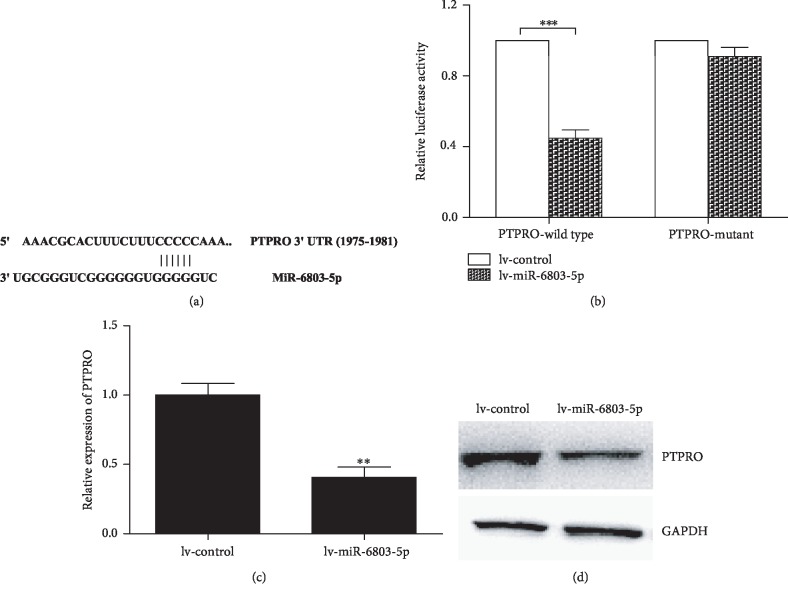
miR-6803-5p targetedly regulated PTPRO in CRC. PTPRO 3′UTR positions recognized by miR-6803-5p; PTPRO was the target of miR-6803-5p (^∗∗∗^*P* < 0.001); reduced PTPRO mRNA in HCT116 cells in contrast to control cells (^∗∗^*P* < 0.01; *n* = 3); reduced PTPRO protein in HCT116 cells in contrast to controls (^∗∗^*P* < 0.01; *n* = 3).

We perform bioinformatics analysis in TargetScan database. miR-6803-5p can specifically combine with PTPRO 3′UTR at 1975-1981 position (Figure 2(a)). Accordingly, it is hypothesized that miR-6803-5p could targetedly regulate PTPRO. The luciferase reporter assay reveals miR-6803-5p can influence its expression by directly targeting PTPRO in CRC cells (Figure 2(b)). As shown in Figures 2(c) and 2(d), the expression of PTPRO is affected in miR-6803-5p-overexpressed cells, which suggests miR-6803-5p can downregulate PTPRO in CRC cells. Taken together, PTPRO is a posttranscriptional regulatory target of miR-6803-5p. Nonetheless, whether miR-6803-5p affect the function of CRC cells remains largely unknown and it has been investigated in the following experiments.

### 3.3. miR-6803-5p Promotes Cancer Cell Proliferation, Invasion, EMT, and Inflammation

**Figure 3:**
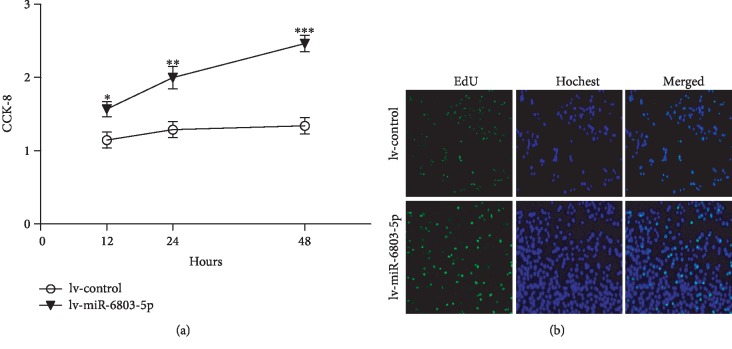
miR-6803-5p promoted the proliferation CRC cells. miR-6803-5p promoted HCT116 cells proliferation assayed by CCK-8 (^∗^*P* < 0.05, ^∗∗^*P* < 0.01, ^∗∗∗^*P* < 0.001; *n* = 3); EdU assay showed miR-6803-5p promoted HCT116 cell proliferation (*n* = 3).

**Figure 4:**
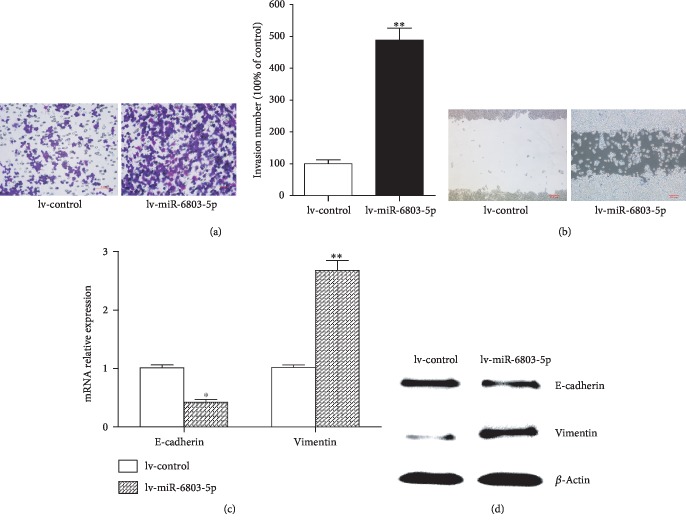
MiR-6803-5p enhanced cancer cell invasion, migration and EMT. High invasion number of HCT116 cells when miR-6803-5p was overexpressed in cells (^∗∗^ *P* < 0.01; *n* = 3); miR-6803-5p promoted HCT116 cells migration estimated by scratch analysis (*n* = 3); miR-6803-5p decreased E-cadherin mRNA but increased Vimentin mRNA expression in HCT116 cells at 12 h (^∗^*P* < 0.05, ^∗∗^*P* < 0.01; *n* = 3); miR-6803-5p reduced E-cadherin but elevated Vimentin protein expression in HCT116 cells at 12 h (*n* = 3).

**Figure 5:**
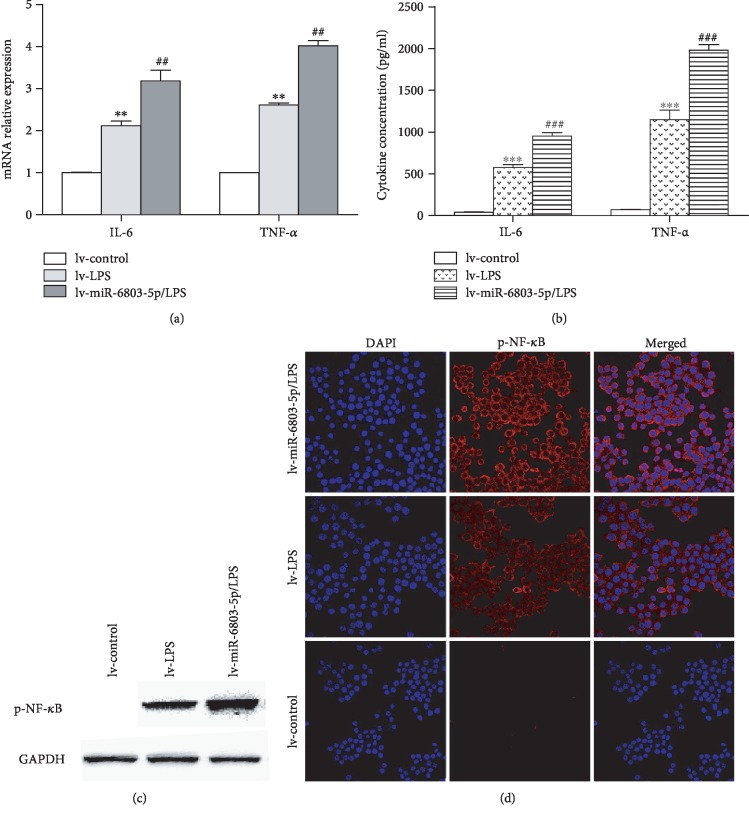
miR-6803-5p aggravated inflammation via NF-*κ*B in HCT116 cells. miR-6803-5p enhanced LPS-induced mRNA expression of cytokines in HCT116 cells (^∗∗^*P* < 0.01 vs. lv-control; ^##^*P* < 0.01 vs. lv-LPS; *n* = 3); miR-6803-5p promoted LPS-induced protein expression of cytokines in HCT116 cells (^∗∗∗^*P* < 0.001 vs. lv-control; ^###^*P* < 0.001 vs. lv-LPS; *n* = 3); miR-6803-5p promoted the expression of phosphorylated NF-*κ*B in cancer cells stimulated by LPS (*n* = 3); miR-6803-5p increased NF-*κ*B activation induced by LPS in cancer cells (*n* = 3).

As evidenced by CCK-8 and EdU assay, cell proliferation is obviously promoted in miR-6803-5p-overexpressed HCT116 cells compared with controls (Figures 3(a) and 3(b)). Besides, as presented in Figure 4(a), the invasion of cancer cells is obviously enhanced in miR-6803-5p-overexpressed HCT116 cells demonstrated by transwell assay. The scratch assay shows miR-6803-5p promotes the migration of CRC cells when it is upregulated in CRC cells (Figure 4(b)). Moreover, the expression of epithelial marker E-cadherin is decreased, while the stromal marker vimentin is increased in miR-6803-5p-overexpressed HCT116 cells (Figures 4(c) and 4(d)). Therefore, miR-6803-5p can contribute to colorectal carcinogenesis by promoting cancer cell proliferation, invasion, migration, and EMT. It is well known that inflammatory cytokines play a vital role in colorectal carcinogenesis, such as IL-6 and TNF-*α*. Results in this study have revealed that miR-6803-5p could enhance the expression of IL-6 and TNF-*α* in CRC cells under simulation of LPS (Figures 5(a) and 5(b)). In addition, LPS activates HCT116 cells and induces activation of NF-*κ*B, while miR-6803-5p further enhances LPS-induced inflammatory cytokine generation in CRC cells by promoting the activation of NF-*κ*B (Figures 5(c) and 5(d)). Taken together, miR-6803-5p enhances LPS-induced inflammation in CRC cells via activating NF-*κ*B, a key transcriptional factor regulating the inflammatory response in cancer cells.

### 3.4. miR-6803-5p Aggravates Inflammation through NF-*κ*B Activation via Targeting PTPRO in CRC Cells

**Figure 6:**
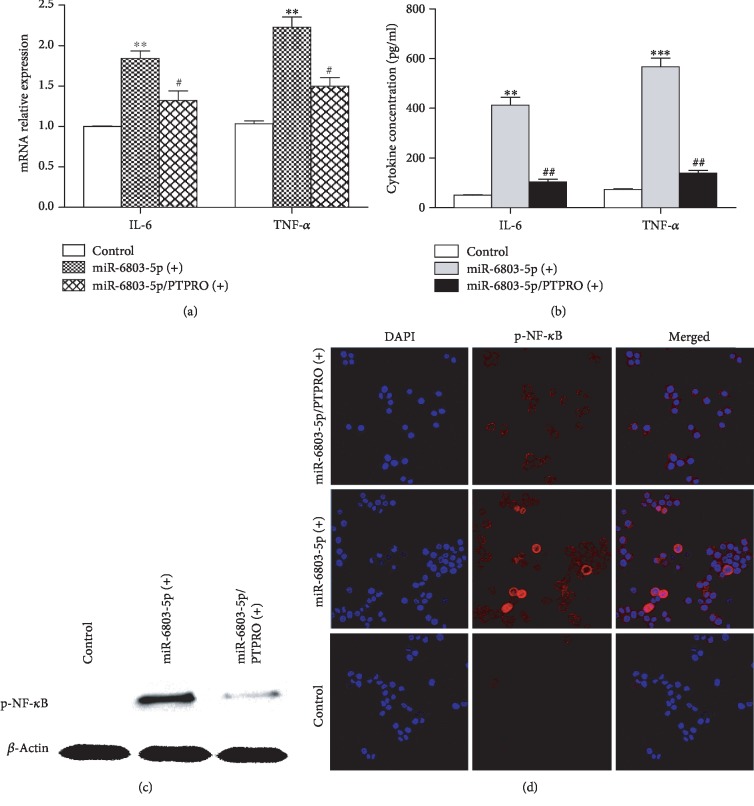
miR-6803-5p regulated cancer inflammation by targeting PTPRO. miR-6803-5p promoted mRNA expression of IL-6 and TNF-*α* by targeting PTPRO in HCT116 cells (^∗∗^*P* < 0.01 vs. control; ^#^*P* < 0.05 vs. miR-6803-5p (+); *n* = 3); miR-6803-5p enhanced IL-6 and TNF-*α* secretion of HCT116 cells via targeting PTPRO (^∗∗^*P* < 0.01, ^∗∗∗^*P* < 0.001 vs. control; ^##^*P* < 0.01 vs. miR-6803-5p (+); *n* = 3); miR-6803-5p elevated p-NF-*κ*B expression by targeting PTPRO in HCT116 cells (*n* = 3); miR-6803-5p promoted NF-*κ*B activation via targeting PTPRO in CRC cells (*n* = 3).

In order to further investigate the association between miR-6803-5p and PTPRO and their functions on CRC cells, we apply lentivirus plasmids to coexpress miR-6803-5p and PTPRO in HCT116 cells. miR-6803-5p overexpression promotes the generation of IL-6 and TNF-*α* in HCT116 cells, whereas the production of inflammatory cytokines is obviously inhibited as suggested by gene compensation experiment via upregulation of PTPRO in CRC cells (Figures 6(a) and 6(b)). Besides, the activation of NF-*κ*B induced by miR-6803-5p is significantly restrained in miR-6803-5p and PTPRO cooverexpressed HCT116 cells (Figures 6(c) and 6(d)). Accordingly, miR-6803-5p can enhance inflammation through NF-*κ*B activation via targeting PTPRO in CRC cells.

## 4. Discussion

The current study has firstly reported that miR-6803-5p is dysregulated in CRC, which can promote cancer cell proliferation, invasion, migration, and EMT. In addition, miR-6803-5p can exaggerate inflammatory response in CRC cells via targeting PTPRO through the activation of NF-*κ*B. Accordingly, miR-6803-5p may serve as an important biological marker for CRC.

miRNAs can regulate various genes at the posttranscriptional level. A number of miRNAs have been established as promising targets for the diagnosis and treatment of CRC [21, 22]. Apart from diagnosis, increasing evidence has demonstrated that miRNAs contribute to drug resistance in CRC, showing evidence to use miRNAs as promising therapeutic targets for CRC patients [23, 24]. Moreover, many dysregulated miRNAs have been established as prognostic markers for CRC [25–27]. Taken together, miRNAs are closely related to the diagnosis, treatment, and prognosis assessment of CRC patients. miRNAs can be stable in circulation. Certain miRNAs are encapsulated in extracellular vesicles, such as microvesicles, exosomes, and apoptotic bodies [20, 28, 29]. Those encapsulated miRNAs can be delivered to targeted cells or tissues by extracellular vesicles and mediate communications between cells. Increasing data have suggested exosomes and exosome-encapsulated miRNAs exert crucial roles in the pathogenesis of CRC [29–31]. A previous study has revealed that exosome-delivered microRNA-375 inhibited the progression and dissemination of CRC cells by targeting Bcl-2 [32]. It has been revealed that decreased expression of exosome-delivered miR-548c-5p is related to CRC prognosis [30]. Accordingly, miRNAs delivered by exosomes are involved in CRC pathogenesis probably by mediating intracellular communications between circulation and local tumor microenvironment. Our previous research has implicated exosome-delivered miR-6803-5p act as a useful marker for the diagnosis and prognosis of CRC [19]. In the current study, we have reported that miR-6803-5p is negatively associated with PTPRO regarding the expression in CRC. Besides, PTPRO is confirmed as a targeted gene of miR-6803-5p. Moreover, the expression of E-cadherin is significantly decreased, while Vimentin is obviously increased when miR-6803-5p is overexpressed in CRC cells at 12 h. As a result, miR-6803-5p can promote CRC cells EMT. In addition, miR-6803-5p is capable of promoting cancer cell proliferation and invasion at 12 h. Accordingly, the enhanced effects of cell invasion and migration are attributed to increased EMT of CRC cells induced by miR-6803-5p. Most interestingly, increased activation of NF-*κ*B and aggravated inflammation in CRC cells have been demonstrated when miR-6803-5p is upregulated in HCT116 cells. All these findings have suggested that miR-6803-5p is involved in CRC by regulating cancer cell growth, invasion, and EMT through NF-*κ*B signaling.

PTPRO belongs to the R3 subtype family and is associated with a number of biological processes. It has been recognized as a promising tumor suppressor in kinds of cancer [4–6]. However, little is known of the relationship between PTPRO and noncoding RNAs, particularly miRNAs in CRC. Several recent studies have elucidated certain miRNAs are associated with PTPRO in kinds of diseases but not cancer, for instance, miR-17-92 and miR-548Cc-5p [33–35]. Our study has initially revealed miR-6803-5p affects CRC cell growth and cancer inflammation by downregulating PTPRO through the activation of NF-*κ*B. As a result, miR-6803-5p/PTPRO can serve as novel targets for CRC. Although we have identified the altered effect of miR-6803-5p on cancer inflammation is dependent on NF-*κ*B, more studies are warranted to elucidate the downstream signaling pathway of miR-6803-5p/PTPRO involved in colorectal carcinogenesis.

To be summarized, this research firstly suggests the vital role of miR-6803-5p in CRC by targeting PTPRO, a well-established tumor suppressor. Thus, the mechanism of miR-6803-5p in carcinogenesis needs to be further investigated to utilize this noncoding miRNA as a tumor oncogene.

## Figures and Tables

**Table 1:** Characteristics of cases and controls in this study.

	CRC (*n* = 95)	Control (*n* = 80)
Age		
< 60 years	40	32
≥ 60 years	55	48
Sex		
Females	41	31
Males	54	49
TNM		
I	20	
II	24	
III	31	
IV	20	
Tumor grade		
High	49	
Low	46	
Lymph node metastasis		
No	36	
Yes	59	
Liver metastasis		
No	58	
Yes	37	

**Table 2:** Primers.

Genes	Forward (5′~3′)	Reverse (5′~3′)
Human E-cadherin	CCAAAGCCTCAGGTCATA	CAGCAAGAGCAGCAGAA
Human Vimentin	ACTTTGCCGTTGAAGCTG	CTCAATGTCAAGGGCCAT
Human TNF-*α*	GTCAACCTCCTCTCTGCCAT	CCAAAGTAGACCTGCCCAGA
Human IL-6	AGTCCTGATCCAGTTCCTGC	CTACATTTGCCGAAGAGCCC
Human PTPRO	TATTGTGAGCCTCCGTGTGT	GCCAAGCCTTTTCAGTGACA
Human GAPDH	AAGGAAATGAATGGGCAGCC	TAGGAAAAGCATCACCCGGA

## Data Availability

Data supporting findings in our study are available from the corresponding authors upon request.
